# Effectiveness and quality of risk communication process in Ethiopia: The case of risk communication during cholera outbreak in Addis Ababa, Ethiopia

**DOI:** 10.1371/journal.pone.0265203

**Published:** 2022-08-19

**Authors:** Nardos Gelana Adera, Bezawit Ketema, Eshetu Girma

**Affiliations:** Department of Preventive Medicine, School of Public Health, College of Health Sciences, Addis Ababa University, Addis Ababa, Ethiopia; Cranfield University, UNITED KINGDOM

## Abstract

**Background:**

WHO states risk communication as the conversation of actual information, guidance, and thoughts between specialists and people fronting risks to their well-being, economic or social safety. As risk communication activities are complex and costly usually, evaluation assessment are the best approach to advance risk communication interventions. This study aims to evaluate the effectiveness of risk communication and the quality of health communication materials developed during the 2019 cholera outbreak at Addis Ababa, Ethiopia.

**Methods:**

A community-based parallel mixed design was conducted from May to June 2020 at Addis Ababa. A total of 605 adults were selected randomly from Addis Ketema sub-city and ten purposely selected adults were interviewed for qualitative data. In addition eight health communication materials on cholera were randomly selected for evaluation. The quantitative data were analyzed using SPSS version 25. After audio recorded interviews transcribed verbatim and translated into English the qualitative data were entered into open code version 4.02 for analysis. And then the data were analyzed using thematic analysis.

**Result:**

Respondents exposed for cholera related messages and outbreak information were 71.8% and 52.7% respectively. Respondents have moderate knowledge for cholera with (M = 14.72 and SD ±4.02) with (0–34) scale range. Both Television and radio spots were found as simple and easy to understand and printed health communication materials score low quality. Poor documentation, lack of data management system and less attention for risk communication activities were identified as a gaps in risk communication in the study settings.

**Conclusion:**

this study revealed the positive effects of risk communication messages in increasing individuals’ knowledge. Even though the risk communication spots were found to be simple and easy to understand, the quality of printed materials was low and less attention was given for the risk-communication activities. Thus, strengthening the quality of risk communication and materials development process is very important to bring desired effects in disease prevention strategies and for effective emergency responses in the future.

## Introduction

Effective risk communication is one of the vital components of outbreak management. WHO states risk communication as the conversation of actual information, guidance, and thoughts between specialists and people fronting risks to their well-being, economic or social safety [1]. The scope of health communication also comprises disease prevention, health protection, promotion, health care strategy, emergency response and improvement of life quality and health of individuals within the community [2]. During outbreak management, health communication interventions are an important component in managing any infectious disease.

Since 1970, different parts of Ethiopia have been recurrently affected with cholera outbreaks [3]. Cholera is an acute intestinal infection which is caused by consumption of contaminated food or water by the bacterium called Vibrio cholera and if not treated it rapidly leads to severe dehydration and death [4]. According to July 2019 WHO report, 688 cases of cholera with 23 confirmed and 15 related deaths have been informed from April to June from five regions in Ethiopia, including Addis Ababa administrative city [5].

Provision of clean drinking water, improved sanitation and personal hygiene are the mainstay of cholera prevention that need to be communicate with the public [6]. A lack of available information during public health emergencies leads to speculations and seeking information from less credible sources which also results in misinformation and rumors. Therefore, having effective communication in place will alleviate this problem to some degree [1].

In the government-led Cholera outbreak response 2019, collaborating with different partners health communication interventions have been done which focused on Hygiene promotion, Social Mobilization, & raising Community awareness [7]. However, evidences are very limited to indicate the health communication aspect in the outbreak management and its effect on behavioral change. Therefore, this study was aimed to evaluate the effectiveness and quality of risk communication process during the 2019 cholera outbreak in Addis Ababa, Ethiopia.

## Methods

### Ethical statement

The study received ethical approval before the data collection from research ethical committee of school of Public Health Addis Ababa University. Then written informed consent was obtained from respondents after clear explanation was given on the purpose, procedure, potential risks and benefits of participating and the right to withdraw from the study at any time throughout their interview. The right of the respondents not to answer some questions or withdraw was respected. Confidentiality of study participants was assured and each interview was conducted with strict privacy.

### Study area

The study was conducted in Addis Ababa, the capital city of Ethiopia. Based on the 2019 United Nations population estimation and projection the city has an estimated 2,757,729 population. The city is divided into ten sub-cities. Addis Ketema sub-city was selected for the study among high cholera cases reported sub-cities during the cholera outbreak in 2019 [7]. Addis Ketema Sub-city Administration has an area of 7.41 sq.km with 271,644 Population. The sub-city is found in the northern part of Addis Ababa and has 10 weredas.

### Study design and period

Community based parallel mixed design was conducted from May-June 2020. This study design included both quantitative and qualitative elements in the same phase of the research process. Even though the data were analyzed independently the results were interpreted together.

### Sample size and sampling procedures

The sample size for quantitative part of the study was determined by single population proportion formula assuming, prevalence of knowledge on cholera to be 50%, marginal error (d) 5% and confidence interval of 95%. 50% has been preferred due to lack of similar studies in Ethiopia. Accordingly, the sample size was calculated to be 384. The final sample size was calculated by adding a design effect of 1.5 and considering 5% non-response rate. Therefore, the final sample size was 605 participants. A multistage random sampling method was used to select study participants. The sample size was allocated proportionally to all selected woredas (the smallest administrative unit) from a total of 4234 households. The sampling frame for each woreda was found from Addis Ketema sub-city administration office and the final households were selected using a systematic random sampling technique. In those households one head of the house (if available) or adults with age ≥ 18 were selected randomly.

For the qualitative part three key informant and seven in-depth interviews were conducted. All participants were purposely selected at various levels. First individuals who have worked on emergency response during cholera outbreak were selected as a key informant from the emergency operation center. Secondly, participants for in-depth interview were selected from the most affected area (sub-city) during the outbreak. Participants were selected purposely from different age group. In addition, five printed health communication materials, two radio spots and one TV spot prepared for cholera outbreak were used for the interview and checklist evaluation.

### Data collection process

Data collection instrument was adapted from similar studies. It included structured interviewer administered questionnaires, check list and interview guides.

The structured questionnaire was used to collect data on socio-demographic and household status, source of health information, exposure of messages and knowledge about cholera among selected adults at household level.

The instrument was pre-tested on 5% of similar population one week before data collection. The interview guides were prepared based on the CDC crisis and emergency risk communications manual (CERC) and used to explore the risk communication process. The checklist was used to evaluate the quality of materials using the modified CDC clear communication index score. The Communication Index provides a set of research-based criteria to develop and assess public communication products. It includes 13 items in four major parts. The index assessed materials in 6 areas which include Main Message, Call to Action, language, Behavioral Recommendations, Numbers and Risk that score out of 6, 2, 2 and 3. The total score was out of 100. Material that score 89 or below shows that the material need improvement.

The quantitative data were collected by face–to-face interview technique by trained data collectors. However, the interviews and material evaluation were conducted by the investigators.

### Data analysis procedure

The quantitative data were coded, checked for clarity and entered into Epi data version 3.1. After that the data were analyzed using SPSS version 25.0. Descriptive statistics were presented with mean, standard deviation, frequency and percentage. Linear regression analysis was used to determine the relationship between the outcome and each independent variables after checking the assumptions and P-value less than 0.05 was used to declare statistical significance. The qualitative data were analyzed thematically and supported by quotations.

## Result

### Background characteristics

Among the total study participants completed response was obtained from 582 participants with the response rate of 96.1%. The mean age of participants was 39.1 with (SD ± 9.86) and more than half of the participants were females (76.1%). One hundred sixty two (28.2%) of them had completed primary school and 206 (35.4%) participants were housewives. (Table 1).

**Table 1:** Socio-demographic characteristics of study participants in Addis Ketema sub-city, Addis Ababa, Ethiopia, 2020.

Variables	characteristics	Frequency *(N = 582)*	Percentage (%)
**Age in years**	20–34	244	41.9
	35–49	261	44.8
	50–64	77	13.2
**Sex**	Male	139	23.9
	Female	443	76.1
**Religion**	Muslim	156	26.8
	Orthodox	272	46.7
	Protestant	127	21.8
	Catholic	27	4.6
**Marital status**	Single	88	15.1
	Married	391	67.2
	Divorced	26	4.5
	Widow	77	13.2
**Educational status**	Unable to read and write	64	11.0
	Able to read and write	116	19.9
	Primary school	164	28.2
	Secondary school	142	24.4
	Technical	57	9.8
	University/higher education	39	6.7
**Occupation**	Government employee	80	13.7
	Private employee	140	24.1
	Merchant	72	12.4
	Daily laborer	21	3.6
	Housewife	206	35.4
	Student	11	1.9
	Other (includes unemployed)	52	8.9

Regarding the household status more than half of the households 299 (51.4%) use shared (public) toilet and 29.4% of them had pit latrine with a cement slab. Functional TV was available in the majority of respondents (96.7%) and 45.4% of them have functional radio.

### Exposure of cholera message

**Fig 1:**
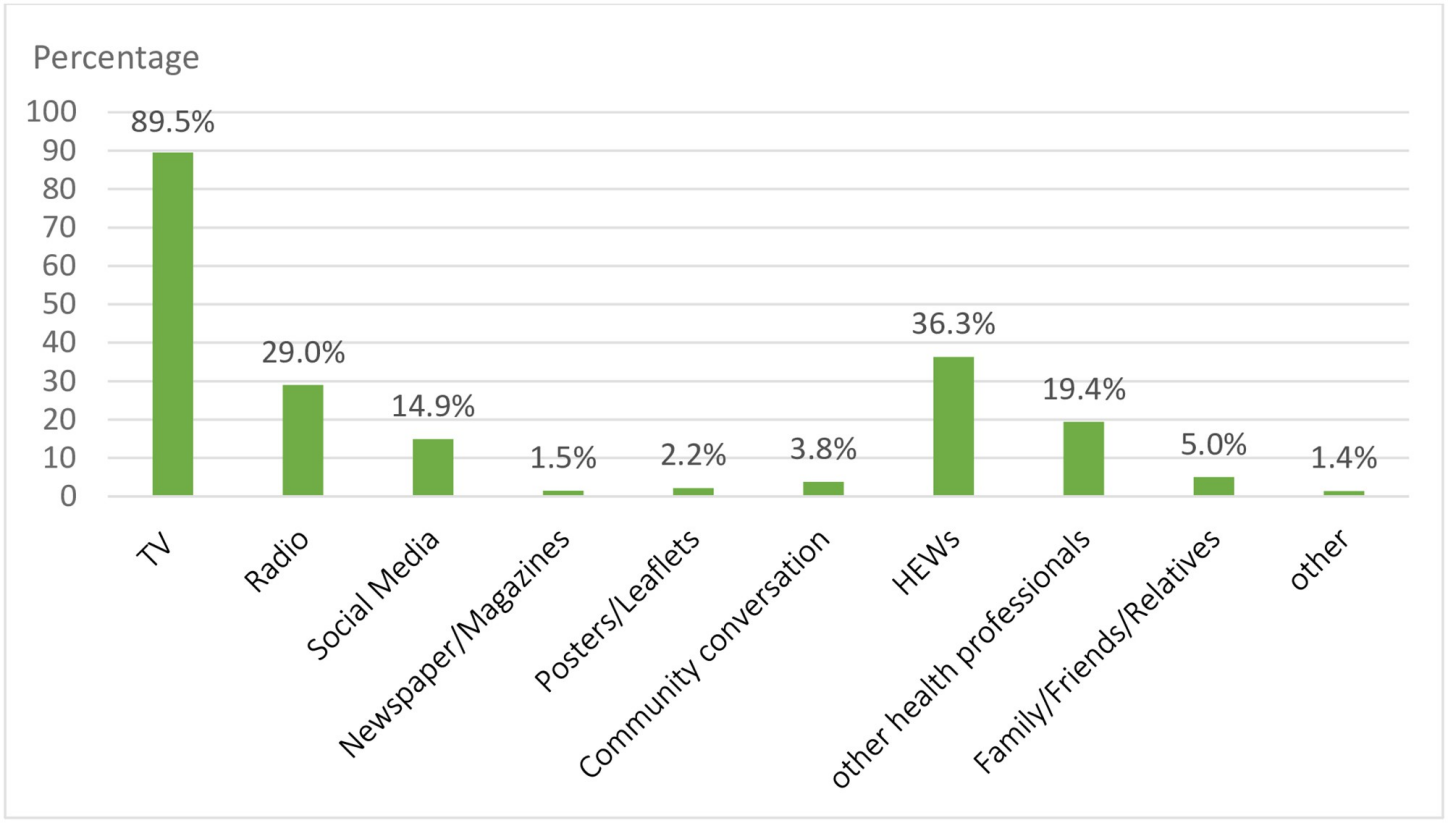
Sources of health information among study participants in Addis Ketema sub-city, Addis Ababa, Ethiopia, 2020. The x-axis shows the different source of health information options and the y-axis shows the percentage given for each selected options. The green color shows the amount of percentage selected by participants for each sources of health information options. The total number of participants were 582.

Among the multiple responses regarding the source of health information TV got the highest rate 521(89.5%) followed by Health extension workers 36.3% and Radio 29.0%. (Fig 1).

From the total of respondents, 418 (71.8%) have been exposed to cholera/AWD messages in the past one year.

The majority of respondents 326 (78%) seen those messages from TV, 45.5% of them got from HEWs and 20.6% of them listened from Radio. The exposure level of messages among respondents watching TV daily was 73.3% and 77.6% for regular radio listeners. The Exposure level was also found to be high among the female respondents (75.8%) compared to males (59.0%).

The study found that 307 (52.7%) of the respondents heard/seen information about the cholera outbreak in Addis Ababa even though they were living in a high cholera-prevalence area with the last outbreak recorded in 2019.

(Table 2) shows that among the transmitted cholera related messages during the outbreak, respondents mostly recall “Proper hand washing” with the highest rate (60.0%) followed by Boiling of water” and “keeping toilet clean” at 50.0% and 25.6% respectively.

**Table 2:** Main messages recalled by respondents in Addis Ketema sub-city, Addis Ababa, Ethiopia, 2020.

Messages	N	Percentage
About proper hand-washing	251	60.0
Boiling of water	209	50.0
Keeping toilet clean	107	25.6
Eating food while it	59	14.1
Cholera causes watery diarrhea and repeated vomiting	44	10.5
Washing vegetables and fruits	39	9.3
Safe food hand	35	8.4
How to use ORS	27	6.5
Seeking health care during symptoms	8	1.9
How to treat diarrhea at home	4	1.0

*Multiple answers possible

### Knowledge on cholera

The overall knowledge score for cholera was moderate with mean 14.72 and SD ±4.02 out of 34 knowledge scores. Among the respondents who correctly answered the causes for cholera, majority of them 60.0% indicated “drinking contaminated water” and 27.8% of them indicated “unhygienic disposal of excreta and refuse” as a cause for cholera. According to descriptive result, majority of them (84.4%) correctly responded that “Watery diarrhea” as a common Symptom for cholera followed by “repeated vomiting” with 74.7%. (Table 3).

**Table 3:** Knowledge items responses on cause and symptoms of cholera among respondents in Addis Ketema sub-city, Addis Ababa, Ethiopia, 2020.

Variable	Items	N*	Percentage (% yes responses)
**Cause of cholera**	Drinking contaminated water	349	60.0
	Unhygienic disposal	162	27.8
	Poor hygiene	150	25.8
	Eating rotten food	87	14.9
	Unwashed fruits and vegetables	52	8.9
	Don’t know	55	9.5
	Other (Rain, cold climate…)	28	4.8
**Symptoms of cholera**	Watery diarrhea	491	84.4
	Repeated vomiting	435	74.7
	Fever	85	14.6
	Tiredness	48	8.2
	Loss of appetite	29	5.0
	Weight loss	24	4.1
	Dry mouth	5	0.9
	Don’t know	34	5.8
	Other (headache, shivering…)	33	5.7

* Multiple responses possible, N = 582

Among the total respondents, 46.7% of them answered the correct answer that cholera can affects everyone where as 36.4% of them stated that cholera affects only children. (Table 4) summarizes the total knowledge scores of participants for each items.

**Table 4:** Summary of knowledge scores among respondents in Addis Ketema sub-city, Addis Ababa, Ethiopia, 2020.

Knowledge Items	Total number of correct items	Mean	Standard deviation	Min	Max	Score Range
Knowledge on cause of cholera	5	1.31	0.833	0	5	5.00
Knowledge on symptoms	2	1.59	0.619	0	2	2.00
Knowledge on MOT	4	0.82	0.859	0	4	3.00
Knowledge on treatment	7	4.09	1.060	0	7	7.00
Knowledge on prevention	9	2.93	0.935	0	9	6.00
Knowledge on susceptibility	1	0.47	0.499	0	1	1.00
Knowledge on severity	6	3.48	1.601	0	6	6.00

Total mean score M = 14.72, SD±4.02, Cronbach’s alpha = 0.689, *N = 582*

### Relationship between knowledge and independent variables

A parametric test is used to see the mean difference between the independent variables with knowledge. According to an independent t-test analysis, there was a statistically significant difference between male and female respondents in mean knowledge scores for cholera, (t (580) = -2.988, P < 0.005, 95% CI = -1.92347, -.39760). The mean values indicate that female respondent had more knowledge on cholera (N = 443, M = 15.0023) than males (N = 139, M = 13.8417). There was also a significant difference in score between the exposed and non- exposed respondents for cholera message with (t (580) = 11.514, P < 0.001, 95% CI = 3.19633, 4.51105). The mean knowledge score among the exposed group (N = 418, M = 15.8110) was higher than the non-exposed (N = 164, M = 11.9573).

### Determinants of knowledge on cholera

Multivariable linear regression analysis revealed that sex of respondents, educational status, occupational status, source of health information, exposure for cholera message and outbreak information were predictors of knowledge on cholera.

Accordingly, knowledge for cholera increased by 1.298 units among female respondents compared to males. The effect of source of health information on knowledge for cholera increased by 1.849 units for those who gets health information from TV and 0.911 units for those who gets health information from HEWs as compared to others sources of health information. The effect of exposure for cholera messages on knowledge for cholera increased by 3.077 units among exposed group compared to non-exposed. Similarly, the effect of exposure for outbreak information on knowledge for cholera increased by 1.644 units among exposed group compared to non-exposed. (Table 5).

**Table 5:** Predictors of knowledge for cholera on multiple linear regression analysis among, respondents in Addis Ketema sub-city, Addis Ababa, Ethiopia, 2020.

Variables (N = 582)	Value	ß	β	P-value	95% CI	
					Lower	Upper
**Age in Years**		-0.007	-0.017	0.713	-0.044	0.030
**Sex**	Male (ref)					
	Female	1.298	0.138	0.001*	0.523	2.074
**Religion**	Catholic (ref)					
	Muslim	0.029	0.003	0.967	-1.332	1.389
	Orthodox	0.065	0.008	0.922	-1.233	1.362
	Protestant	0.565	0.058	0.417	-0.803	1.933
**Marital status**	Married (ref)					
	Single	-0.044	-0.004	0.925	-0.968	0.879
	Divorced	-0.112	-0.006	0.865	-1.406	1.182
	Widowed	-0.731	-0.062	0.103	-1.610	0.149
**Educational status**	Unable to read & write (ref)					
	Able to read & write	0.347	0.035	0.489	-0.638	1.333
	Primary	1.186	0.133	0.020*	0.186	2.187
	Secondary	2.670	0.285	P<0.001*	1.554	3.786
	Technical	2.306	0.171	0.001*	0.979	3.632
	Higher/university	2.926	0.182	0.001*	1.281	4.571
**Occupational status**	Gov’t employee (ref)					
	Private employee	-1.204	-0.128	0.009*	-2.104	-0.303
	Merchant	-1.778	-0.146	0.001*	-2.861	-0.694
	Daily laborer	-1.515	-0.070	0.066	-3.129	0.099
	Housewife	-1.154	-0.137	0.014*	-2.074	-0.234
	Student	-1.43	-0.048	0.182	-3.531	0.674
	Other	-1.963	-0.139	0.001*	-3.130	-.797
**Functional TV**	Yes	1.360	0.060	0.126	-0.385	3.104
	No (ref)					
**Functional radio**	Yes	0.047	0.006	0.893	-0.644	0.739
	No (ref)					
**Source of health information**	Television	1.849	0.141	P<0.001*	0.830	2.869
	Radio	0.057	0.006	0.878	-0.674	0.789
	Social media	0.774	0.069	0.169	-0.330	1.877
	Newspaper/magazine	1.397	0.043	0.209	-0.785	3.579
	Poster/Leaflets	0.372	0.014	0.684	-1.423	2.168
	Community conversation	0.836	0.040	0.239	-0.558	2.230
	HEWs	0.911	0.109	0.006*	0.256	1.566
	Other health professionals	0.658	0.065	0.064	-0.038	1.354
	Friends/family	0.636	0.034	0.306	-0.584	1.855
**Frequency of listening radio**	Everyday	0.218	0.018	0.657	-0.743	1.179
	Two or more days per week	0.934	0.063	0.087	-0.136	2.004
	Once a week	0.315	0.028	0.493	-0.588	1.218
	Less than once a week	0.893	0.043	0.212	-0.511	2.297
	Not at all (ref)					
**Frequency of using Social media**	Everyday	0.398	0.035	0.504	-0.772	1.569
	Two or more days per week	0.518	0.039	0.333	-0.532	1.567
	Once a week	1.123	0.060	0.088	-0.168	2.415
	Less than once a week	1.377	0.020	0.545	-3.084	5.838
	Not at all (ref)					
**Exposure for cholera message**	Yes	3.077	0.344	P<0.001*	2.420	3.733
	No (ref)					
**Exposure for outbreak information**	Yes	1.644	0.204	P<0.001*	1.058	2.231
	No (ref)					

ß = Unstandardized regression coefficient, β = Standardized regression coefficient

*Statistically significant = p< 0.05, ref. = Reference category

R^2^ = 0.445, Adjusted R^2^ = 0.403, F change = 10.570, P = <0.001

### Quality of cholera spots

#### Participant’s socio-demographic characteristics

Study Participants were adults who were living in Addis ketema sub-city. A total of seven participants who were selected purposively were interviewed and asked about their opinion on spots prepared for cholera prevention. One TV spot and two radios spots were used to evaluate participants reflection and their understanding towards the communication materials.

**Table:** 

Participants’ characteristics		Number
**Age**	20–39	5
	40–59	2
**Sex**	Male	3
	Female	4
**Educational Status**	Secondary	3
	Diploma and above	4
**Occupation**	Student	2
	Private employee	3
	Government employee	1
	Housewife	1
**Marital Status**	Single	3
	Married	4

Almost all in-depth interview participants reported that the message was about cholera prevention and the importance of keeping personal hygiene. Participants also reported that the messages transmitted on the spots were clear and easy to understand. A 33yrs old female participant said,

“*The message is about cholera and the main thing it says that when cholera occurs we should go to the health center*…”

Regarding self-involvement few participants reflected that the messages were targeted for those people who are living in poor hygienic area. A 52yrs old female participant said,

“…*I think it is prepared for us especially for those who are living in this area*, *because mostly the disease affects a community like this*. *Because we are living in a very sophisticated environment*. *I think it is for us*.”

Related with attraction of message almost all of the participants declared that they liked the spots and messages transmitted. Most of them mentioned that they were attracted to the messages and how the messages were presented. However, when participants asked about the acceptance of materials most of the respondents mentioned that the tone of the message was so fast. For instance, a 39yrs old male participant said,

“*The first thing is the way of its presentation*. *For example*, *it is fast and maybe some peoples can’t hear it at fast*. *And the second one is I think it has some kind of classical music and thus it make it difficult to hear the message and I don’t think it will be helpful to get the main concept too*.”

When assessing the call to action from the materials almost all of the respondents reported that, the message recommended them to prevent themselves from cholera. Mostly, they mentioned that the message is asking them to keep hygiene, proper hand washing, and also to seek immediate health care when they have diarrhea and other symptoms. For instance, a 20yrs old female participant said,

“… *I understand how to prepare a home solution with 6 spoon sugar*, *lemon*, *and water if someone is affected by diarrhea and vomiting*, *and when ORS is not available*. *And also we should go to the health center to see a Doctor while taking the solution*.”

### Quality of printed health communication materials

Five randomly selected printed heath communication materials (2 leaflet, 2 poster and 1 banner) prepared for cholera/AWD prevention were evaluated using a checklist. The materials were prepared for the general public by MOH, EPHI and other partners together. The checklist assessed the materials based on the revised CDC clear index score sheet which used to assess public communication materials. It contains four parts namely: Main message call to Action and language, behavioral recommendation, numbers and risk that score out of 6, 2, 2 and 3. The total score was out of 100. Material that score 89 or below shows that the material need improvement.

Based on the checklist evaluation the main message that most of the materials transmitted was about cholera disease and its prevention methods. The main messages was written on the top and end section of the materials even though not all of the materials had identified main message content specifically. The materials include more than one calls to action for the target audiences.

Both the main messages and call to action on the materials were presented in the active voice and words that target audiences’ use. But some of the words used on the materials were not familiar with the general public (target audience) and can make them confused. Most of the materials included more than one behavioral recommendations to prevent cholera and to reduce its transmission like proper hand washing, eating food while it is hot, washing vegetables and fruits thoroughly, using proper and clean toilet, to use clean and safe drinking water and the like. But in most of the materials the importance of this behavioral recommendations was not explained very well.

Most of the materials used bullets and number list for the recommended behaviors. And all of the materials didn’t use decimals, fractions and percentages. Related with presenting the risk most of the materials tried to explain the nature of the problem that cholera is infectious disease and an emergency case that need immediate treatment and health care. Based on the CDC index score sheet all materials score below 89 which needs improvement. (See S1 Data).

### Risk communication process

Key informants who were working in the risk-communication team during cholera outbreak (2019) were interviewed about their experience on the whole risk-communication process. The findings were summarized with six sub-categories. Three participant were interviewed including one team coordinator and two team members from the risk-communication unit.

#### Risk communication system

An emergency operation center (EOC) was launched by Ethiopian public health institute in 2016. The national risk-communication and community engagement technical group involved different professionals both from the government and other partners including health education professionals, media professionals and other social science professionals.

“*Well*, *within the team there are media professionals*, *public relation professionals*, *health education and promotion experts*, *and also there are professionals from WHO and UNICEF emergency health communication development programs*.”

*(KII 3*: *Team coordinator)*

#### Preparation during the outbreak

The risk-communication unit was working based on an emergency preparedness response plan (EPRT) which was prepared at the national level. During the cholera outbreak (2019) the main objectives of the risk-communication activities were creating awareness about cholera and enabling the community to prevent themselves from the outbreak. A team coordinator said as follow,

“*The first objective was to empower the community and enabling them to interact with protective behaviors*. *So that*, *they will be engaged and take responsibility to protect themselves*.”

During message development information for inputs were gathered from the surveillance team and by doing rapid assessments on the affected areas. And after identifying the preferred source of information in the community they have prepared revised materials. However, the communication materials were not pretested within the community due to the emergency situation. One team member said that,

“…*after we draft the messages there will be In-house pre-testing*. *And sometimes we made a pre-test*, *but in situations like this due to the emergency we did In-house pre-testing like discussing with the technical working group*. *Then*, *we sum up the ideas and used them*. *We haven’t done a full pre-testing due to the emergency*.”

*(KII 1*: *Team member)*

#### Message dissemination process

Prepared messages and other reports related to the outbreak was disseminated to the public using different approaches. The common way for transmitting the messages was sharing daily updates and new information using daily brief on radio and TV news. To ensure trust and credibility among the community information were announced early for the public using news and other media platforms. As the key informant said,

“…*yeah communicating first is the main thing in risk-communication*. *So*, *information and reports were combined and delivered early and daily at the time*. *Especially*, *every week on our press-release detailed information was transmitted on the media for the public and we tried to be trusted sources of information*.”

Participant’s also reflected that they used all media to transfer their messages by giving trainings and orientation on how to communicate risk to the public. Both government and private channels were involved on message dissemination in A.A and at regional level. Channel preference assessment was also done at A.A to identify the media choice of peoples during the outbreak.

“*Almost we used all media especially those with high coverage and audience including private channels*. *For example here in A*.*A children and their caregivers were choose Kana TV as their preferred channel*. *So*, *like this*, *we identified the channels and used media mix to display our messages*.”

*(KII 3*: *Team coordinator)*

It was also mentioned that the high cost for broadcasting messages and using prime time were challenges on messages delivery.

#### Community engagement

The risk communication team was also working on engaging the community on message preparation and other interventions using different social networks.. In Addis Ababa, they were mainly focused on the most affected sub-cities. They tried to figure out the root cause of the outbreak in those sub-cities to take appropriate interventions. A team member said,

“…*we have conducted a rapid assessment on two selected sub-cities*. *Then*, *when we see the root causes for the outbreak*, *there was food contamination in some area*. *I remember the case for Addis Ketema sub-city was due to the Ramadan season many people were fasting and foods were prepared and distributed at the same time in the area*. *So due to that*, *the case was shooting rapidly*. *And when we see the case of Akaki sub city it was shooting due to contaminated river water*. *Some peoples used that water and transmitted for others*. *So*, *with the identified root causes*, *we tried to design interventions specifically*.”

Beside awareness creation, the team was also involved in providing service for the community. For instance they were empowering the community by availing services like water treatment chemicals and giving health education at household levels. Participants also mentioned that they prepared media monitoring team to scan false information and rumors.

“*We had a media monitoring team that monitors the media*, *ministry media*, *and also social media*. *So*, *after the team identified the misinformation then*, *we will identify its channel that reaches many people and we were announcing them during our press-conference for the public*. *And also there was a surveillance team on the affected areas that monitor rumors working with community HEWs*. *So*, *by using their information we were drafting and preparing messages*.”

*(KII 1*: *Team member)*

#### Monitoring and evaluation system

The planning and monitoring team in the unit was working on documenting daily activities, reports and other communications using those documented files for reporting and review meetings. Lack for a data server and not documenting lesson learned stories regularly in every outbreaks were mentioned as the main problem in their documentation and data management system. Participants also mentioned that even though they planned to evaluate risk-communication activities at the end, it was not done as expected.

“…*evaluation was done for the cholera response as a whole*. *When we say evaluation we evaluated the success of every region like lessons learned and what were the challenges and the like*. *But as I know specifically we didn*’*t evaluate the impact of risk-communication and whether it was successful or not*.”

*(KII 1*: *Team member)*

The overloading of tasks and the occurrence of other outbreaks were mentioned as reasons for not during the evaluation timely.

#### Challenges and way forward

The risk-communication team faced many challenges during such emergency response activities. Some of the challenges on the risk-communication activities were related to bureaucracy problems and financial issues. A team member commented that,

“*Yeah*….*there was a bureaucracy problem*. *Normally during other times*, *we were bidding tender to produce prepared audio and video spots and we have been facing financial problems every time due to the bureaucracy*. *And again when we came to this emergency*, *such problems limited our speed to reach the community as quick as possible because of the challenges we faced to produce and disseminate our materials*.”

In addition to this, the less attention given for risk-communications, considering risk-communication as a public relations, and insufficient human-power in the unit were other challenges faced during the emergency response.

“*Yeah*, *one of our big challenge was*, *there was a misunderstanding of Risk-communication among the decision-makers*. *And naturally*, *it is a backbone for any emergency response but in practice*, *less attention was given for Risk-communication activities and this makes us to work more advocacy on it*.”

*(KII 3*: *Team coordinator)*

To overcome those challenges the unit tried to engage partners for mobilizing resources and they were giving orientations for media and public relation professionals about risk-communication and community engagement. Participants also suggested some way forwards related to risk-communication approach and future responses.

“…*the first thing that we should focus on is documentation and data formatting*. *Documentation was an overlooked activity every time which underestimates our work*. *When an outbreak occurs everybody will try to get those data in his way and even there was a time that we started from scratch*. *And for these reasons*, *whenever an emergency happened peoples who engaged in previous outbreaks will be called or the new person will start to organize the team again*. *Therefore*, *there should be a regular reporting system and strong documentation system for risk communication activities nationally because it has many impacts on data quality and emergency response*.”

*(KII 2*: *Team member)*

## Discussion

Effective risk-communication is the most valuable element in public health emergency responses and it should cover both the risks and actual health problems [8]. This study revealed that, exposure for cholera related message and outbreak information in the past one year was 71.8% and 52.7% respectively. This implies that the risk-communication messages related to the outbreak information were communicated less compared to cholera prevention messages.

The finding of this study showed that, 78% of the respondents exposed for cholera messages from Television and TV was the most preferred source of health information. The qualitative finding also stated, that rapid assessments were done on channel preference in selected sub-cities. Public TV channels with high coverage rate were used to transmit the risk-communication messages during the outbreak. Selecting the right and most referred communication channels is necessary on delivering risk-communication messages effectively [9].

Evidences suggest that television and radio are the most broadly used mass-media and immediate channels of communication during an emergency [9]. Most of the risk-communication messages and programs during the outbreak were transmitted through TV using press-conference and other health related TV shows. This finding is in lined with the study conducted at Haiti that television was the most preferred forms of communication for receiving cholera messages [10]. Therefore, this indicates that it is necessary to design relevant health communication messages and resources using TV for the general public.

The outcome of effective risk communication also includes increasing awareness and knowledge among target audiences [11]. This study shows that, respondents have moderate knowledge about cholera and its prevention. The finding was higher than the finding of the study conducted in Tanzania [12]. This knowledge difference may be due to the effect of the risk-communication activities done in this specific area and the recent history of the outbreak in the study area.

Even though most of the communication materials and spots were prepared on cholera prevention methods, it was found that respondents have low knowledge on cholera prevention methods. Majority of them didn’t indicated more than two cholera prevention methods. 67.1% of the respondents stated proper hand washing as cholera prevention and the finding was comparable with study conducted in Haiti [10]. Therefore, the communication materials have missed the main elements to be communicated to the public to prevent the disease [6].

The mean score knowledge for cholera was high among female respondents than males. This discrepancy may be due to the fact that most of the females are housewives and can spent more time obtaining health information from mass media such as TV and radio. This finding is also seen in the post-outbreak study conducted in Iran [13]. Even though it was unable to evaluate the separate communication effect on cholera knowledge, the easy access for health information showed a significance effect in increasing the public knowledge.

As education is primarily important and related to knowledge it was found that educational status of respondents has a significant difference in cholera knowledge. Respondents with higher educational level had better knowledge on cholera compared to Illiterates. This finding is also align with a study conducted in Bangladesh [14].

Knowledge for cholera increased by 3.077 among exposed respondents than non-exposed. This could be due to the additive effect of exposure of messages on existing knowledge. It is also reported that in qualitative result that participant’s gain new information from the spots and most of them remembered that they exposed to the spots and easily identified the main messages. The outcome of message exposure on knowledge also seen in other studies [15, 16].

Risk-communication requires to be carefully planned, applied and combined with emergency management activities and processes [17]. The study found that, a separate communication plan was derived for Addis Ababa from the national communication plan and messages were prepared after doing rapid assessments on affected areas. This will help to identify key problems in the areas and to set priorities in the message development process. The implication of risk-assessment and identifying community risk-perception for successful emergency response was seen in study conducted in Liberia [18].

As emergencies are time-sensitive, communicating information quickly is crucial. So that, the first source of information usually becomes the preferred source [19]. In the present study, it is reported that daily updates and new information related to the outbreak were delivered early for the public using daily briefs and press-releases and the unit tried to be trusted source of information. Trust and source of information were seen as essential factors for risk-communication to be reliable and effective in other studies [20].

The international Crisis and Emergency Risk communication (CERC) manual stated community engagement as a strategy to understand the cultural context of the community and to build trust in developing materials during emergencies [19]. The study found that the risk communication unit tried to engage community leaders and other influential person during mass-education. It was also mentioned that beside awareness creation and health education activities, availing sanitation services and materials for the community were supplied collaborating with other government agencies. Such activities may help to build trust and to increase the credibility of the organizations among the community.

The International Health Regulations (2005) indicated that all WHO Member states to develop risk communication capacities as a core capacities and states those capacities to regularly assessed and evaluated through external evaluation [21]. Finding from this study shows that, even though evaluation is planned for the risk communication activities it was not done at the end of the outbreak. Incorporating evaluation results and feedbacks from partners and communities will help to improve ongoing and future emergency responses.

The bureaucracy problems including lack of attention for the risk communication activities, lack of enough budget for material production and lack of professionals in the field were the major challenges for the risk communication process. It was also found that, the problem with poor documenting and data management system influenced and affect the risk communication work. A study conducted in Liberia showed that the successful risk communication strategies including various risk communication approaches helped for effective disease control during Ebola outbreak [18]. Strengthening risk communication with community involvement activities will bring successful and effective impact in the overall emergency responses.

The result of the clear index score shows that, the quality of the communication materials score is low. This indicates that, the materials need improvement and further need assessments. Most of the materials didn’t incorporate enough information about the disease and its prevention methods. This gap is also reflected from participant that, they need more clarification and detail information on the disease from the cholera spots.

Most of the communication materials and spots were found to be clear and easy to understand. However, using unfamiliar words on the messages and problems on spot presentations were found to be a problem. The use of inappropriate words, and problems on message designs also seen on other evaluation studies [22, 23].

## Conclusion

The study revealed that the effect of risk communication messages in increasing individuals’ knowledge. Even though the cholera spots were found to be simple and easy to understand the quality of printed materials were low and less attention given for the risk-communication activities affect the effectiveness of the risk-communication process. Thus, we recommend to strength risk communication and materials development process since it is very important to bring desired effects in disease prevention strategies and for effective emergency responses in the future.

## Supporting information

S1 File (SAV)

S1 Data CDC clear index score sheet result. (DOCX)

## Abbreviations

AWD: Acute watery diarrhea
CDC: Communicable disease control
CERC: Crisis and emergency risk communication
EPHI: Ethiopian public health institute
ERC: Emergency risk communication
HEWs: Health extension workers
IHR: International Health Regulation
MOH: Ministry of health
RCCE: Risk communication and community engagement
WHO: World health organization
