# Supplementary material for: Effectiveness and quality of risk communication process in Ethiopia: The case of risk communication during cholera outbreak in Addis Ababa, Ethiopia
Source: PLoS One. 2022 Aug 19;17(8):e0265203. doi: 10.1371/journal.pone.0265203 (PMC9390904; doi:10.1371/journal.pone.0265203)
Supplement: S1 Data — (DOCX) [file pone.0265203.s002.docx]

Annex: CDC clear index score sheet result

**Checklist assessment for material 1: (Leaflet about cholera disease)**

|  | Questions | Score | |
| --- | --- | --- | --- |
|  | Part A: Core | Yes (1) | No (0) |
|  | **Main message and call to Action** |  |  |
| 1 | Does the material contain one main message statement? |  | 0 |
| 2 | Is the main message at the top, beginning, or front of the material? |  | 0 |
| 3 | Does the material include one or more calls to action for the primary audience? | 1 |  |
|  | **Language** |  | |
| 4 | Do both the main message and the call to action use the active voice? | 1 |  |
| 5 | Does the material always use words the primary audience uses? | 1 |  |
| 6 | Is the most important information the primary audience needs summarized in the first paragraph or section? |  | 0 |
|  | Part A score | Total 3 /6 | |
|  | **Part B: Behavioral recommendation** |  |  |
| 7 | Does the material include one or more behavioral recommendations for the primary audience? | 1 |  |
| 8 | Does the material explain why the behavioral recommendation(s) is important to the primary audience? | 1 |  |
|  | Part B score | Total 2 /2 | |
|  | **Part C: Numbers** |  |  |
| 9 | Does the material always present numbers the primary audience uses? | 1 |  |
| 10 | Does the audience have to conduct mathematical calculations? (here yes scores “0” and No scores as “1”) |  | 1 |
|  | Part C score | Total 2 /2 | |
|  | **Part D: Risk** |  |  |
| 11 | Does the material explain the nature of the risk? | 1 |  |
| 12 | Does the material address both the risks and benefits of the recommended behaviors? | 1 |  |
| 13 | If the material uses numeric probability to describe risk, is the probability also explained with words or a visual? |  | 0 |
|  | Part D score | Total 2 /3 | |

**Calculation for the score:** Part A=3/6 Part B=2/2 Part C =2/2 Part D =2/3

Total Score =9/13*1OO=69.2

**Checklist assessment for material 2: (Leaflet about AWD)**

|  | Questions | Score | |
| --- | --- | --- | --- |
|  | Part A: Core | Yes (1) | No (0) |
|  | **Main message and call to Action** |  |  |
| 1 | Does the material contain one main message statement? | 1 |  |
| 2 | Is the main message at the top, beginning, or front of the material? | 1 |  |
| 3 | Does the material include one or more calls to action for the primary audience? | 1 |  |
|  | **Language** |  | |
| 4 | Do both the main message and the call to action use the active voice? | 1 |  |
| 5 | Does the material always use words the primary audience uses? | 1 |  |
| 6 | Is the most important information the primary audience needs summarized in the first paragraph or section? |  | 0 |
|  | Part A score | Total 5 /6 | |
|  | **Part B: Behavioral recommendation** |  |  |
| 7 | Does the material include one or more behavioral recommendations for the primary audience? | 1 |  |
| 8 | Does the material explain why the behavioral recommendation(s) is important to the primary audience? | 1 |  |
|  | Part B score | Total 2 /2 | |
|  | **Part C: Numbers** |  |  |
| 9 | Does the material always present numbers the primary audience uses? | 1 |  |
| 10 | Does the audience have to conduct mathematical calculations? (here yes scores “0” and No scores as “1”) |  | 1 |
|  | Part C score | Total 2 /2 | |
|  | **Part D: Risk** |  |  |
| 11 | Does the material explain the nature of the risk? | 1 |  |
| 12 | Does the material address both the risks and benefits of the recommended behaviors? | 1 |  |
| 13 | If the material uses numeric probability to describe risk, is the probability also explained with words or a visual? |  | 0 |
|  | Part D score | Total 2 /3 | |

**Calculation for the score:**

Part A=5/6 Part B=2/2 Part C =2/2 Part D =2/3

Total Score =11/13*1OO=84.6

**Checklist assessment for material 3: (Poster about cholera prevention methods)**

|  | Questions | Score | |
| --- | --- | --- | --- |
|  | Part A: Core | Yes (1) | No (0) |
|  | **Main message and call to Action** |  |  |
| 1 | Does the material contain one main message statement? | 1 |  |
| 2 | Is the main message at the top, beginning, or front of the material? | 1 |  |
| 3 | Does the material include one or more calls to action for the primary audience? | 1 |  |
|  | **Language** |  | |
| 4 | Do both the main message and the call to action use the active voice? | 1 |  |
| 5 | Does the material always use words the primary audience uses? | 1 |  |
| 6 | Is the most important information the primary audience needs summarized in the first paragraph or section? | 1 |  |
|  | Part A score | Total 6 /6 | |
|  | **Part B: Behavioral recommendation** |  |  |
| 7 | Does the material include one or more behavioral recommendations for the primary audience? | 1 |  |
| 8 | Does the material explain why the behavioral recommendation(s) is important to the primary audience? |  | 0 |
|  | Part B score | Total 1 /2 | |
|  | **Part C: Numbers** |  |  |
| 9 | Does the material always present numbers the primary audience uses? | 1 |  |
| 10 | Does the audience have to conduct mathematical calculations? (here yes scores “0” and No scores as “1”) |  | 1 |
|  | Part C score | Total 2 /2 | |
|  | **Part D: Risk** |  |  |
| 11 | Does the material explain the nature of the risk? |  | 0 |
| 12 | Does the material address both the risks and benefits of the recommended behaviors? |  | 0 |
| 13 | If the material uses numeric probability to describe risk, is the probability also explained with words or a visual? |  | 0 |
|  | Part D score | Total 0 /3 | |

**Calculation for the score:**

Part A=6/6 Part B=1/2 Part C =2/2 Part D =0/3

Total Score = 9/13*1OO=69.2

**Checklist assessment for material 4: (Poster about water treatment)**

|  | Questions | Score | |
| --- | --- | --- | --- |
|  | Part A: Core | Yes (1) | No (0) |
|  | **Main message and call to Action** |  |  |
| 1 | Does the material contain one main message statement? | 1 |  |
| 2 | Is the main message at the top, beginning, or front of the material? | 1 |  |
| 3 | Does the material include one or more calls to action for the primary audience? | 1 |  |
|  | **Language** |  | |
| 4 | Do both the main message and the call to action use the active voice? | 1 |  |
| 5 | Does the material always use words the primary audience uses? | 1 |  |
| 6 | Is the most important information the primary audience needs summarized in the first paragraph or section? | 1 |  |
|  | Part A score | Total 6 /6 | |
|  | **Part B: Behavioral recommendation** |  |  |
| 7 | Does the material include one or more behavioral recommendations for the primary audience? | 1 |  |
| 8 | Does the material explain why the behavioral recommendation(s) is important to the primary audience? |  | 0 |
|  | Part B score | Total 1 /2 | |
|  | **Part C: Numbers** |  |  |
| 9 | Does the material always present numbers the primary audience uses? | 1 |  |
| 10 | Does the audience have to conduct mathematical calculations? (here yes scores “0” and No scores as “1”) |  | 1 |
|  | Part C score | Total 2 /2 | |
|  | **Part D: Risk** |  |  |
| 11 | Does the material explain the nature of the risk? | 1 |  |
| 12 | Does the material address both the risks and benefits of the recommended behaviors? |  | 0 |
| 13 | If the material uses numeric probability to describe risk, is the probability also explained with words or a visual? |  | 0 |
|  | Part D score | Total 1 /3 | |

**Calculation for the score:**

Part A=6/6 Part B=1/2 Part C =2/2 Part D =1/3

Total Score = 10/13*1OO=76.9

**Checklist assessment for material 5: Banner about cholera prevention**

|  | Questions | Score | |
| --- | --- | --- | --- |
|  | Part A: Core | Yes (1) | No (0) |
|  | **Main message and call to Action** |  |  |
| 1 | Does the material contain one main message statement? | 1 |  |
| 2 | Is the main message at the top, beginning, or front of the material? | 1 |  |
| 3 | Does the material include one or more calls to action for the primary audience? | 1 |  |
|  | **Language** |  | |
| 4 | Do both the main message and the call to action use the active voice? | 1 |  |
| 5 | Does the material always use words the primary audience uses? | 1 |  |
| 6 | Is the most important information the primary audience needs summarized in the first paragraph or section? |  | 0 |
|  | Part A score | Total 5 /6 | |
|  | **Part B: Behavioral recommendation** |  |  |
| 7 | Does the material include one or more behavioral recommendations for the primary audience? | 1 |  |
| 8 | Does the material explain why the behavioral recommendation(s) is important to the primary audience? |  | 0 |
|  | Part B score | Total 1 /2 | |
|  | **Part C: Numbers** |  |  |
| 9 | Does the material always present numbers the primary audience uses? |  | 0 |
| 10 | Does the audience have to conduct mathematical calculations? (here yes scores “0” and No scores as “1”) |  | 1 |
|  | Part C score | Total 1 /2 | |
|  | **Part D: Risk** |  |  |
| 11 | Does the material explain the nature of the risk? | 1 |  |
| 12 | Does the material address both the risks and benefits of the recommended behaviors? |  | 0 |
| 13 | If the material uses numeric probability to describe risk, is the probability also explained with words or a visual? |  | 0 |
|  | Part D score | Total 1 /3 | |

**Calculation for the score:**

Part A=5/6 Part B=1/2 Part C =1/2 Part D =1/3

Total Score = 8/13*1OO=61.5
